# Supplementary material for: High-performance thermomagnetic generator controlled by a magnetocaloric switch
Source: Nat Commun. 2023 Aug 9;14:4811. doi: 10.1038/s41467-023-40634-x (PMC10412618; doi:10.1038/s41467-023-40634-x)
Supplement: Supplementary file 1 — Supplementary Information [file 41467_2023_40634_MOESM1_ESM.pdf]

Supplementary Information for

High-Performance Thermomagnetic Generator Controlled by a Magnetocaloric Switch

Xianliang Liu,[a] Haodong Chen,[a] Jianyi Huang,[a] Kaiming Qiao,[a] Ziyuan Yu,[a] Longlong Xie,[a] Raju V. Ramanujan,[b] Fengxia Hu,[c, d, e] Ke Chu,[f] Yi Long,[a] and Hu Zhang,[a,*]

[a] School of Materials Science and Engineering, University of Science and Technology Beijing, Beijing 100083, P R China.

[b] School of Materials Science and Engineering, Nanyang Technological University, Singapore 639798, Singapore.

[c] Beijing National Laboratory for Condensed Matter Physics, Institute of Physics, Chinese Academy of Sciences, Beijing 100190, P R China.

[d] School of Physical Sciences, University of Chinese Academy of Sciences, Beijing 100049, P R China.

[e] Songshan Lake Materials Laboratory, Dongguan, Guangdong 523808, P R China.

[f] School of Materials Science and Engineering, Lanzhou Jiaotong University, Lanzhou 730070, P R China.

[*]Corresponding author at: School of Materials Science and Engineering, University of Science and Technology Beijing, Beijing, 100083, China. Tel.: +86-10-62333733.

E-mail address: zhanghu@ustb.edu.cn

Supplementary Note 1. The magnetic properties of MCS

As benchmark magnetocaloric material, Gd has a Curie temperature T_C around room temperature and an excellent magnetocaloric effect (MCE). Therefore, Gd metal was selected as the working material for the thermomagnetic generator (TMG) in this work. Supplementary Fig. 1a shows the temperature dependence of zero-field-cooling (ZFC) and field-cooling (FC) magnetization (**M**) for Gd under 0.2 T. It can be found that Gd is ferromagnetic at low temperatures. With the increase in temperature, the Gd experiences a magnetic transition from ferromagnetic (FM) to paramagnetic (PM) states. The Curie temperature T_C, defined as the minimum value of $d\mathbf{M}/dT$ curve as shown in the inset, is 292 K. Moreover, the ZFC and FC curves overlap with each other perfectly without thermal hysteresis, suggesting the nature of second-order magnetic transition (SOMT).

Supplementary Fig. 1b shows the isothermal magnetization curves (**M**-**H**) of Gd. It can be seen that the magnetization at low temperatures increases rapidly at low fields and tends to be saturate with the increase of magnetic field, corresponding to the typical FM state. With the increase of temperature, the magnetization isotherm gradually shows a linear increase with the magnetic field, indicating the PM behavior. In addition, no magnetic hysteresis is observed for all **M**-**H** curves during the field increasing and decreasing modes, confirming the characteristic of SOMT. It should be pointed out that reversible magnetic transition of SOMT is desirable to MCE and TMG since it would not lead to a large discrepancy in the working temperatures during the heating and cooling cycles. Based to the **M**-**H** curves, the magnetic entropy change ΔS_M values for different magnetic field changes were calculated by using Maxwell relation $\Delta S_M(T,H) = \mu_0 \int_0^{\mathbf{H}} \left(\partial M / \partial T\right)_{\mathbf{H}} d\mathbf{H}$, as shown in Supplementary Fig. 1c. The maximum ΔS_M of Gd are 3.14 J/kg K, 5.34 J/kg K, and 7.12 J/kg K for the magnetic field changes of 1 T, 2 T, and 3 T, respectively. The relatively large MCE indicates that the potentially good TMG performance of Gd metal.

According to the Faraday's law, large induced power not only depends on the large $d\Phi/dT$, but also requires a high temperature change dT/dt. Accordingly, the TMG material should also have a small heat capacity (C_P) and large thermal conductivity (λ). Supplementary Figure 1d shows the temperature dependence of C_P in zero field for Gd. The C_P reaches the

peak of ~276.3 J/kg K at T_C, and then decreases gradually with the increase of temperature. The C_P of Gd metal is lower than those of other typical room temperature MCE materials, such as La(Fe, Si)$_{13}$-based alloys[1-3] and NiMn-based Heusler alloys[4, 5]. Besides, the λ of Gd increases from ~9.4 W/m K near 288 K to ~12.2 W/m K near 353 K[6], which is significantly higher than those of other typical MCE materials, such as $Gd_5(Si, Ge)_4$ alloys[7] and MnAs[7]. This low C_P and high λ of Gd metal are favorable to acquiring high TMG performance.

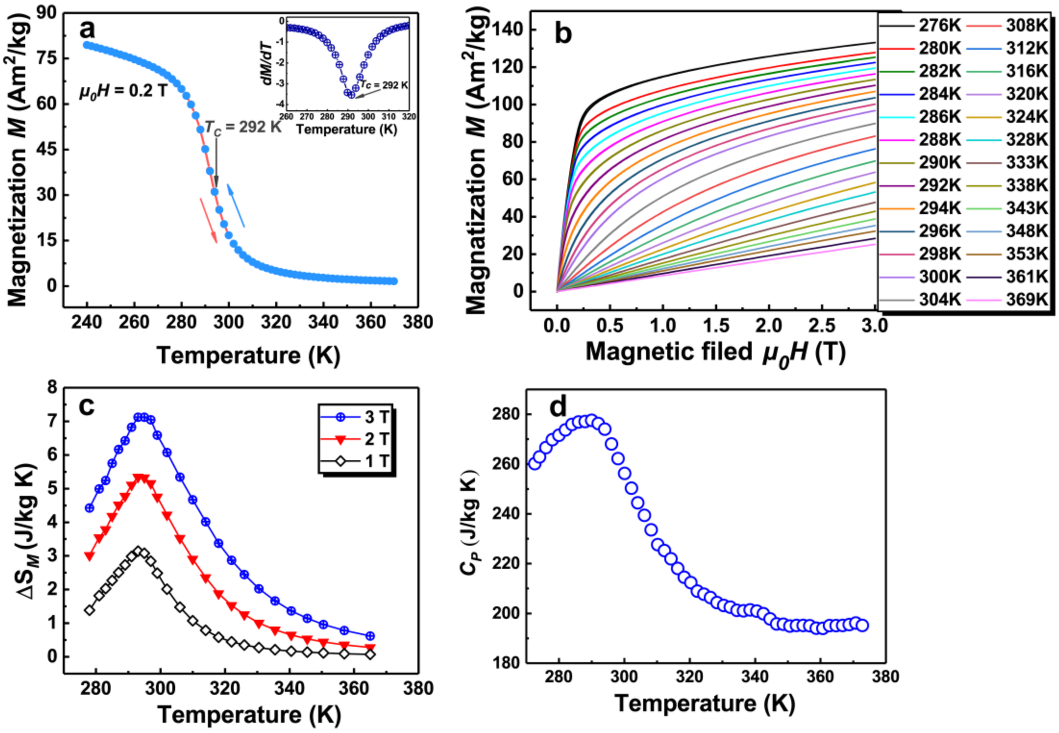

Supplementary Figure 1: Properties test of thermomagnetic material Gd. **a**, Variation curve of magnetization **M** with temperature *T*. The inset shows the magnetization change rate *d***M**/*dT* curve. **b**, Variation curve of magnetization **M** with magnetic field μ_0**H** for different temperatures. **c**, Variation curve of magnetic entropy change ΔS_M with temperature *T*. **d**, Temperature dependence of C_P in zero field. (Source data are provided as a Source Data file.)

Supplementary Note 2. Simulation model of the TMG

To understand the working principle and optimize the structure of the magnetic circuit, we use the finite element simulation to establish a multiphysics model by using the COMSOL Multiphysics software (Version 5.4). Both heat transfer module and magnetic field module were used to analyze the temperature change of the thermomagnetic material (TMM) and the magnetic flux change in the magnetic circuit. A detailed discussion on the numerical model

is described in the following subsections.

(1) Governing Equations for Simulation Models

The key of numerical simulation is to solve the governing equations. Among them, the heat diffusion equation is used to describe the heat transfer process of the MCS in the magnetic circuit, and its expression is:

$$\rho C_P \frac{\partial T}{\partial t} - \nabla \cdot (\lambda \nabla T) = Q \tag{1}$$

In the formula, ρ, C_P, λ, and T are the density, specific heat capacity, thermal conductivity, and temperature of the MCS, respectively. The Q represents the heat absorbed or released by the material when it comes in contact with the fluid. The temperature variation of the MCS in the magnetic circuit is obtained by solving the above equation.

Using Maxwell's equations to describe the transient change of the magnetic field in the magnetic circuit:

$$\nabla \cdot D = \rho \tag{2}$$

$$\nabla \cdot \mathbf{B} = 0 \tag{3}$$

$$\nabla \times E = -\frac{\partial \mathbf{B}}{\partial t} \tag{4}$$

$$\nabla \times \mathbf{H} = J + \frac{\partial D}{\partial t} \tag{5}$$

where D is the displacement current density, ρ is the free charge density, $\mathbf{B}$ is the magnetic flux density, E is the electric field intensity, $\mathbf{H}$ is the magnetic field intensity, J is the conduction current density, and t is the time. Equation (2) describes the Gaussian law for electric fields, and the electric field flux across an arbitrary closed surface is proportional to the amount of charge enclosed within it. Equation (3) describes Gauss's law for magnetic field. Since the magnetic field lines are all closed, the flux contribution to a closed curve is zero. Equation (4) describes Faraday's law of electromagnetic induction, that is, the induced electric field is proportional to the change rate of the magnetic flux. Equation (5) describes Ampere's law that the magnetic field strength is proportional to the change rate of the electric field flux. The magnetic field module obtains the distribution of the magnetic field and the magnetic flux change in the magnetic circuit by solving Maxwell's equations.

(2) Boundary conditions and initial conditions

Supplementary Fig. 2 presents the numerical simulation model of the magnetic circuit. In the numerical model, the magnetic circuit is surrounded by an air domain. A magnetic insulation boundary condition is applied to the edge of the air domain. It represents the boundary where the tangential magnetic field vanishes to zero and can be expressed as:

$$n \times \mathbf{H} = 0 \tag{6}$$

where n and $\mathbf{H}$ represent the normal vector and magnetic field strength of the boundary, respectively.

For the heat transfer module, the cold side temperature is 283 K and the hot side temperature is 363 K. The ambient temperature of 293.15 K is considered as the initial value for all domain temperatures except the hot and cold ends. Meanwhile, the boundary conditions for the regions in the TMG are set as thermal insulation except the sample. This boundary condition means that there is no heat flux across the boundary.

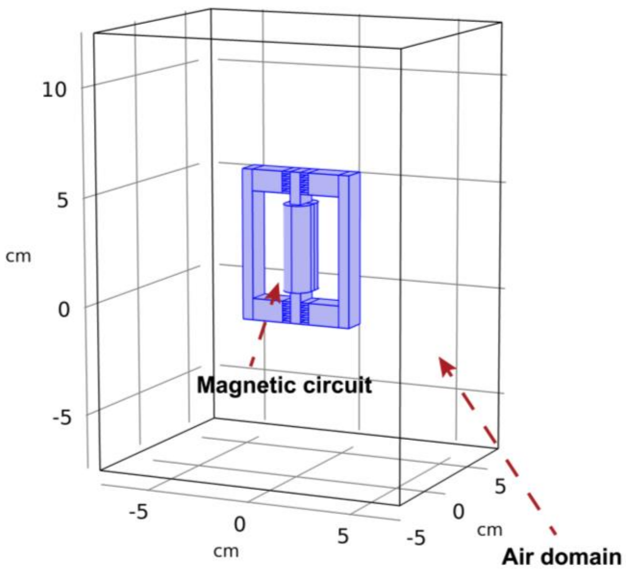

Supplementary Figure2: Numerical simulation model of the magnetic circuit

(3) Mesh independence test

To ensure the accuracy of the numerical model and minimize the computation time, mesh-independent tests were performed using free tetrahedral mesh elements. Extra-coarse (6294 mesh elements), coarser (9862 mesh elements), coarse (14575 mesh elements), normal (22697 mesh elements), fine (44561 mesh elements), and finer (93428 mesh elements) were

tested. Supplementary Fig. 3 shows the simulated induced peak voltage of the TMG as a function of the number of mesh elements. It is found that induced peak voltage reaches saturation when the number of mesh elements reaches 44561. Therefore, we selected fine mesh elements for the subsequent simulation, because it would ensure the calculation accuracy and also reduce the computing resources and time as much as possible.

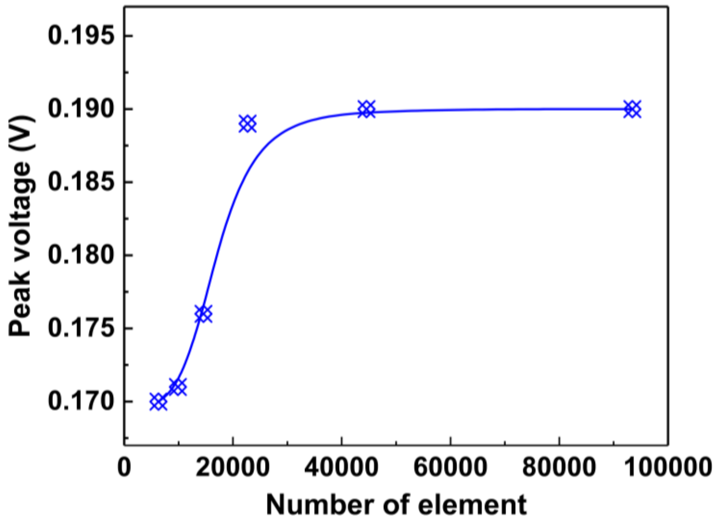

Supplementary Figure3: Simulated induced peak voltage of the TMG as a function of the number of mesh elements. (Source data are provided as a Source Data file.)

Supplementary Note 3. Optimization simulation of the TMG

In order to compare the performance of our TMG design with flux reversal and conventional TMG design without flux reversal, we compare the flux distributions of these two magnetic circuits based on finite element simulations. Five Gd plates with length × width × thickness of 4 × 12 × 1 mm were used in both simulation. Supplementary Fig 4a, b show the simulated magnetic flux distribution of the conventional TMG topology with TMM at (a) FM state and (b) PM state. In the initial state, the cold end is in contact with the TMM, and the temperature of the TMM is lower than T_C and is at FM state. As shown in Supplementary Fig. 4a, the TMM at FM state is magnetized by the permanent magnet and its magnetic flux density **B** is ~0.37 T (light green). The magnetic force line propagates through the TMM to the right yoke. Conversely, when the TMM is heated to PM state, the magnetic flux density **B** of the TMM is reduced to ~0.18 T (dark blue), as shown in Supplementary Fig. 4b. A big

part of magnetic flux propagates to the air domain, resulting in the reduction of the magnetic flux in the right yoke. However, a high residual magnetic flux density remains in the yoke, resulting in a limited magnetic flux change $\Delta\Phi$ in the magnetic circuit. Supplementary Fig. 4c shows the simulated magnetic flux curve during heating and cooling process. The magnetic flux change $\Delta\Phi$ of the traditional magnetic circuit during the cycle is 2.13×10^{-5} Wb.

Supplementary Fig. 4d, e show the simulated magnetic flux distribution of our novel TMG topology with MCS at (d) FM state and (e) PM state. When cold water flows through the left circuit while hot water flows through the right circuit (Supplementary Fig. 4d), the left MCS is at FM state while the right one is at PM state, and so the left circuit is switched ON while the right one is turned OFF. In this case, the magnetic flux flows clockwise in the left circuit. When the hot and cold water are turned over as shown in Supplementary Fig. 4e, the left circuit is switched OFF while the right one is turned ON, and so the magnetic flux flows in the right circuit. It is clearly seen that the magnetic flux in the center yoke is reversed when the hot and cold water are alternated. Supplementary Fig. 4f shows the simulated magnetic flux curve during heating and cooling process. It is clearly seen that the magnetic flux in the induction coil wound around the center yoke change between the negative and positive maximum value, similar to the effect of the pretzel-like topology[1]. In comparison with the conventional TMG design in which the flux only changes between zero and maximum value, the present topology results in nearly twice magnetic flux change $\Delta\Phi$ (4.30×10^{-5} Wb), and thus would increase the induced power by a factor of four. In addition, a small step appears in the magnetic flux curve during each heating/cooling process. Because the T_C = 292 K of Gd is closer to the cold end temperature of 283 K rather than the hot end temperature of 363 K, the FM-PM magnetic transition would occur earlier during the heating than the PM-FM transition during the cooling. This fact suggests that the MCSs on both sides do not turn ON/OFF at the same time even the hot and cold valves are switched simultaneously. Therefore, it causes the step changes of magnetic flux as well as successive induced V peaks. Moreover, it is noted that this topology not only has all the advantages of the pretzel-like topology, such as flux reversal, higher maximum flux density in iron yoke, avoiding the magnetic stray fields and hysteresis effect, but also overcomes the key drawback of the pretzel-like topology, that is, nearly half part of the flux change does not flow through

the induction coil and so it will not contribute the power generation. In our TMG, all the magnetic flux in the conductive magnetic circuit is utilized, which means that the power generated by present topology would be four times the induced power in pretzel-like topology, as the induced power is proportional to the square of the magnetic flux change[8, 9].

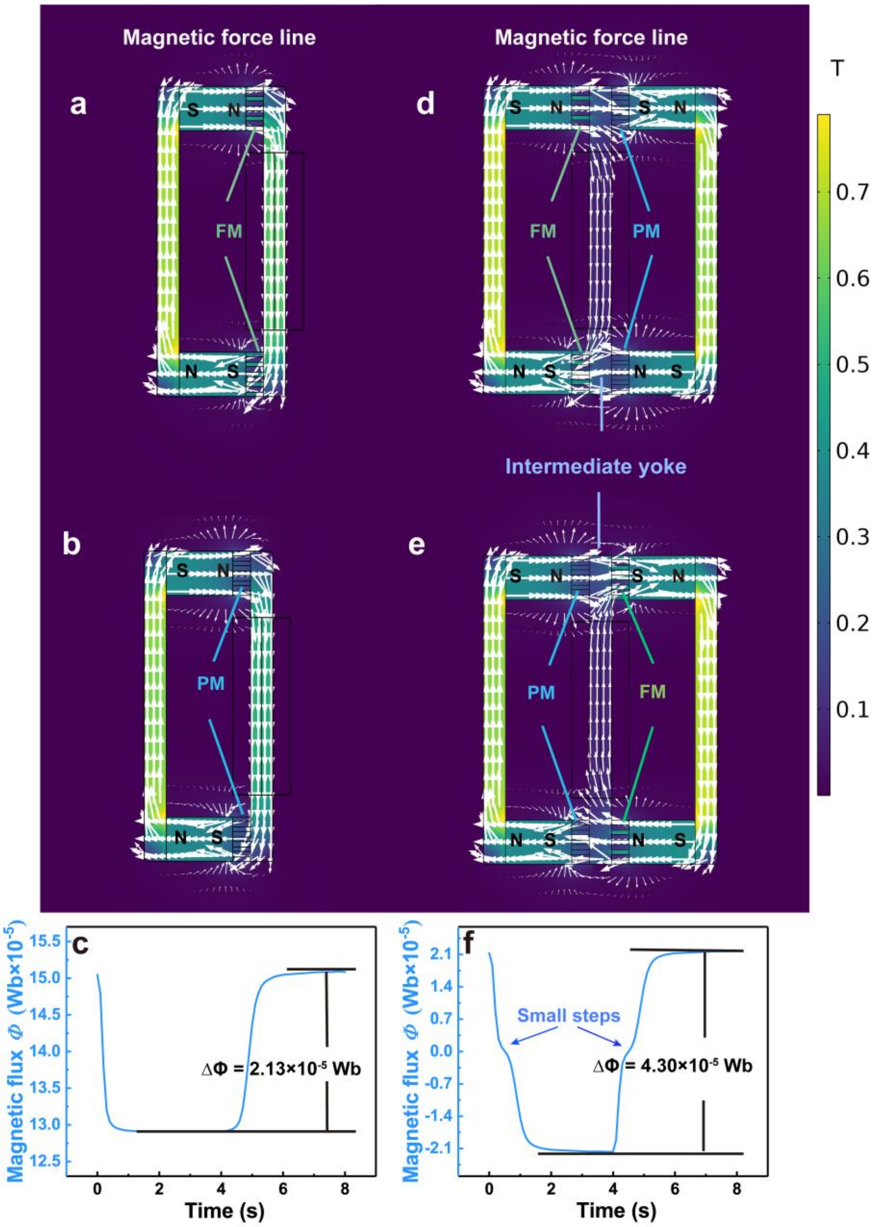

Supplementary Figure 4: Simulation results of two different magnetic circuits. **a**, **b** The distribution of magnetic flux density **B** of the traditional magnetic circuit. **c**, The magnetic flux curve of the traditional magnetic circuit. **d**, **e** The distribution of magnetic flux density **B** of our novel magnetic circuit. **f**, The magnetic flux curve of our novel magnetic circuit. (Source data are provided as a Source Data file.)

(1) Effect of MCS thickness

According to the Faraday's law, the induced electrodynamic potential (V) is determined by the change rate of magnetic flux ($d\Phi/dt$) in a N turns of coil[10, 11]. Moreover, the $d\Phi/dt$ is not just related to the design of magnetic flux topology, but also strongly affected by the structure parameters, such as the thickness and length of TMM. Therefore, we constructed the magnetic circuit of the TMG using finite element simulation, and studied the effect of the structural parameters on the TMG performance in order to select the optimal parameters. Two Gd plates with length × width × thickness of 10 × 12 × 1 mm were used as each MCS in the initial simulation. Supplementary Fig. 5a shows the T-t curves of MCS with different thickness. It is clearly seen that the temperature changes slower with the increase of MCS thickness, e.g., the time for temperature rising from 283 K to ~361 K increases from 1.5 s to 3 s with the MCS thickness increasing from 1 mm to 4 mm. The peak temperature change rate dT/dt of MCS decreases almost linearly from 137.0 K/s to 58.8 K/s with the increase of thickness from 1 mm to 4 mm, as shown in Supplementary Fig. 5b. It suggests that the thicker thickness would lower the dT/dt, thus is not favorable to the TMG. Supplementary Fig. 5c shows the Φ-t curves of MCS with different thickness. As the thickness of the MCS increases from 1 mm to 4 mm, the magnetic flux change $\Delta\Phi$ in the magnetic circuit increases linearly from 1.86×10^{-5} Wb to 6.0×10^{-5} Wb. It is noted that, although the increase of thickness would enhance the $\Delta\Phi$, thicker MCS plate would also lower the temperature change rate dT/dt. Considering that $\frac{d\Phi}{dt}=\frac{d\Phi}{dT}\cdot\frac{dT}{dt}$, larger $d\Phi/dT$ with the increase of thickness results in the increase of $d\Phi/dt$ at first, and then the decrease of dT/dt becomes more predominant with further increasing the thickness, leading to the inflection point and the gradual decrease of $d\Phi/dt$, as shown in Fig. 2c. This result suggests that it is important to balance the magnetic flux change $\Delta\Phi$ and the temperature change rate dT/dt for optimal TMG performance.

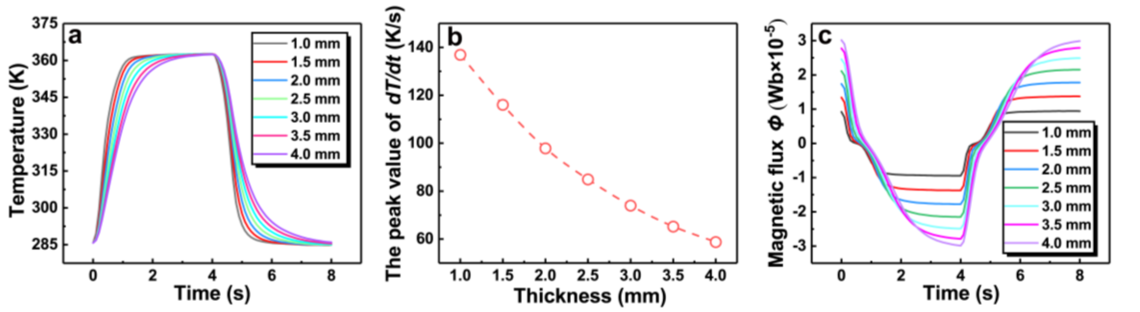

Supplementary Figure 5: The effect of MCS thickness on the magnetic circuit. **a**, The temperature change curve of the MCS. **b**, The peak value of temperature change rate *dT/dt* of the MCS with the different thickness. **c**, The change curve of the magnetic flux in the magnetic circuit with time. (Source data are provided as a Source Data file.)

Based on above results, it is better to make the MCS thinner while ensuring the same cross-sectional area to increase the overall *dT/dt*. Furthermore, we compared the performance of two 2.5 mm thick samples in each sample cabin and five 1 mm thick samples in each sample cabin, as shown in Supplementary Fig. 6. Supplementary Fig. 6a shows the *T*-*t* curves of these two cases. The decrease of thickness increases the *dT/dt*, i.e., the time for temperature rising from 283 K to ~361 K reduces from 2.2 s to 1.5 s. In the case of the same cross-sectional area, the magnetic flux change $\Delta\Phi$ in the magnetic circuit remains unchanged. Due to the increase of *dT/dt*, the magnetic flux change rate $d\Phi/dt$ increases (Supplementary Fig. 6b). Supplementary Fig. 6c compares the peak value of the magnetic flux change rate $d\Phi/dt$ in the two cases, and it can be seen that the peak value of $d\Phi/dt$ increases from 5.9×10^{-5} Wb/s (2.5 mm × 2) to 7.8×10^{-5} Wb/s (1 mm × 5). This result suggests that layered thin plates are favorable to higher TMG performance.

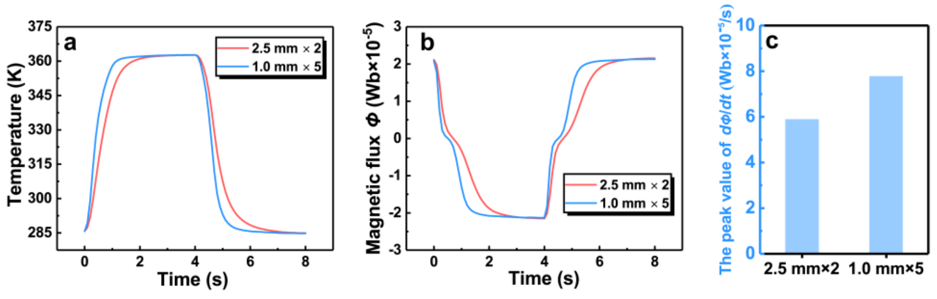

Supplementary Figure 6: Simulation results for two cases of 2.5 mm × 2 and 1 mm × 5. **a**, Temperature change curve. **b**, Magnetic flux change curve. **c**, Peak value of magnetic flux change rate *dΦ/dt*. (Source data are provided as a Source Data file.)

(2) Effect of MCS length

By keeping the five 1 mm thick plates in the sample cabin, the effect of the MCS length on the magnetic flux is further studied by simulation. Supplementary Fig. 7a shows the *Φ*-*t* curves of MCS with different length. The initial *Φ* in the magnetic circuit gradually increases from 0.97×10^{-5} Wb to ~2.28×10^{-5} Wb with the MCS length increasing from 1 mm to 5~7 mm, and then the initial *Φ* starts to decrease with the further increase of MCS length. Correspondingly, the magnetic flux change Δ*Φ* and peak *dΦ/dt* increases largely with the length increasing from 1 mm to 5~7 mm, and then starts to decrease gradually with a further increase in length, as shown in Supplementary Fig. 7b.

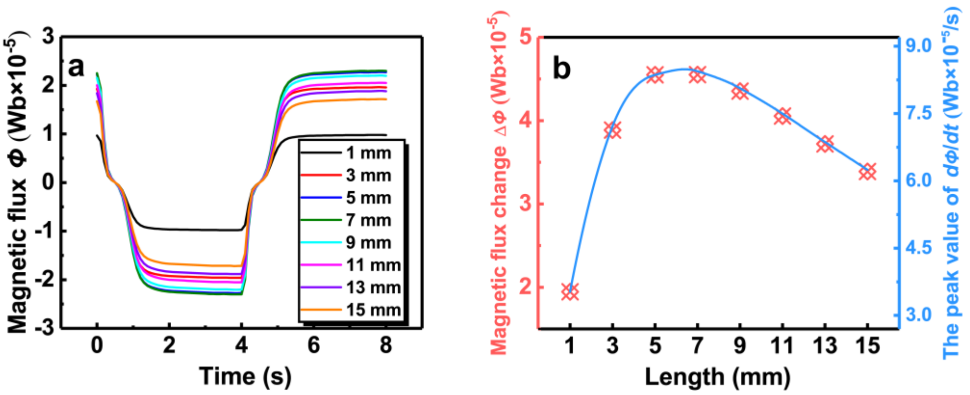

Supplementary Figure 7: The effect of MCS length on the magnetic circuit. **a**, The change curve of the magnetic flux in the magnetic circuit with time. **b**, The magnetic flux change Δ***Φ*** and the peak value of the magnetic flux change rate *dΦ/dt* with the length. (Source data are provided as a Source Data file.)

In order to understand the variation of Δ*Φ*, the magnetic flux density distributions diagram for different length of MCS are simulated as shown in Supplementary Fig. 8. When the length of MCS is 1 mm, such short distance between the permanent magnet and the center yoke could cause a large amount of flux to flow through the right MCS even it is at PM state, i.e., the magnetic flux densities **B** of the left and right MCSs are 0.4 T (ferromagnetic state) and 0.26 T (paramagnetic state), respectively. Thus, a large *Φ* of 5.9×10^{-5} Wb circulates along the outer loop, while only a small *Φ* of 0.97×10^{-5} Wb flows through the center yoke. With the increase of length, it is harder to magnetize the right MCS at PM state, and so more magnetic flux is shunted from the outer loop to the center yoke, resulting in the distinct increase of *Φ* through the center yoke. It is seen that almost no flux flows through the right

circuit when the length reaches 7 mm, indicating that the right MCS is completely OFF. Thus, the Δ*Φ* reaches the maximum value of ~4.55×10^{-5} Wb (Supplementary Fig. 7b). However, when the length is larger than 5~7 mm, the permanent magnet cannot fully magnetize the left MCS, and a large amount of magnetic flux is strayed into the air. So the *Φ* in the magnetic circuit decreases gradually. Supplementary Fig.8h shows that the magnetic flux density *B* of the left and right MCS is 0.15 T (FM state) and 0.06 T (PM state) when the length is 15 mm. Since the thickness remains at 1 mm, the heat transfer from the surface to the core will not be changed, and so the *dT/dt* of the MCS keeps constant. Then, the *dΦ/dt* is only affected by the magnetic flux change Δ*Φ*, and thus the variation of *dΦ/dt* is perfectly consistent with that of Δ*Φ*, as shown in Fig. 2e.

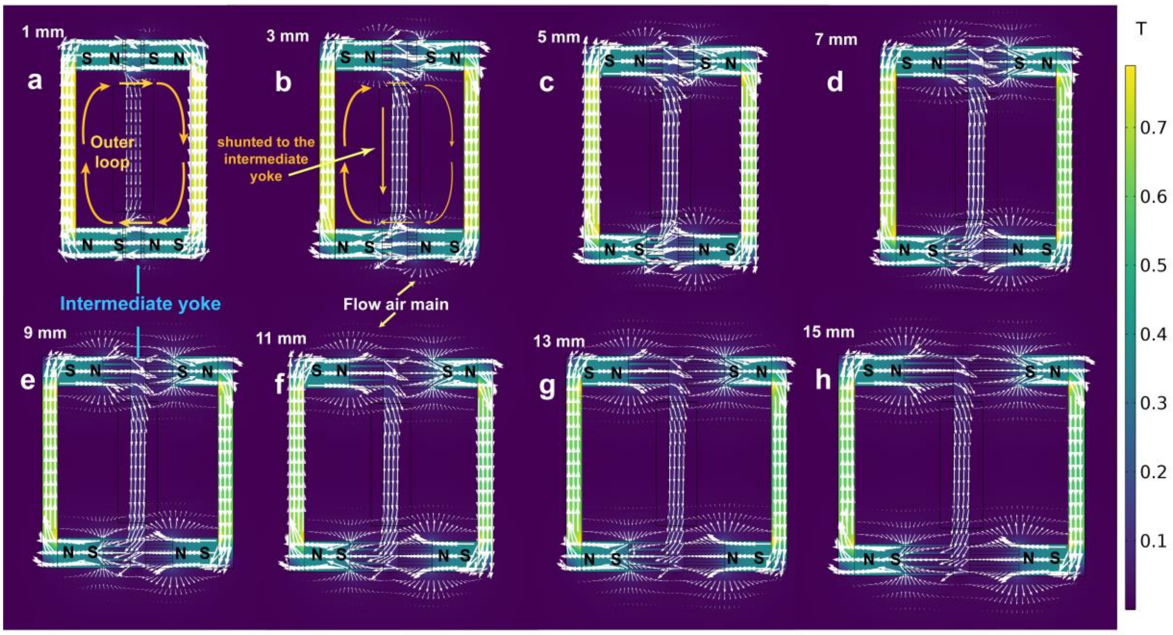

Supplementary Figure 8: The magnetic flux density distribution of the magnetic circuit under different lengths of the MCS. The white arrow is the flow direction of the magnetic flux. **a**, 1 mm. **b**, 3 mm. **c**, 5 mm. **d**, 7 mm. **e**, 9 mm. **f**, 11 mm. **g**, 13 mm. **h**, 15 mm.

(3) Effect of coil turns

Finally, we studied the effect of coil turns on the magnetic circuit performance. Supplementary Fig. 9a shows the schematic diagram of the coil turns. We keep the height *h* of the coil unchanged, and the coil is wound outwards along the length *l* during the winding process. Supplementary Fig. 9b shows the magnetic flux linkage *NΦ* (the magnetic flux in the coil multiplied by the number of turns) as a function of time for MCS with different coil turns.

As the coil turns gradually increase from 600 to 1600, the initial $N\Phi$ increases from 1.36×10^{-2} Wb to 3.56×10^{-2} Wb. Then, the initial $N\Phi$ does not increase significantly with the further increase of coil turns. The magnetic flux linkage change $\Delta N\Phi$ and the peak $dN\Phi/dt$ value as the function of the coil turns are obtained as shown in Supplementary Fig. 9c. It is seen that both $\Delta N\Phi$ and $dN\Phi/dt$ increase almost linearly with the N increases from 600 to 1400, and then tend to become saturation of 7.38×10^{-2} Wb and 13.38×10^{-2} Wb/s with further increasing N higher than 1600. It is found from Supplementary Fig. 9a that the surface coil is about ~2 cm away from the center yoke when $N > 1600$, and so the surface coils might not be able to effectively sense flux changes in the center yoke, resulting in the saturation of $\Delta N\Phi$ and $dN\Phi/dt$. Therefore, 1600 is chosen as the optimized coil turns.

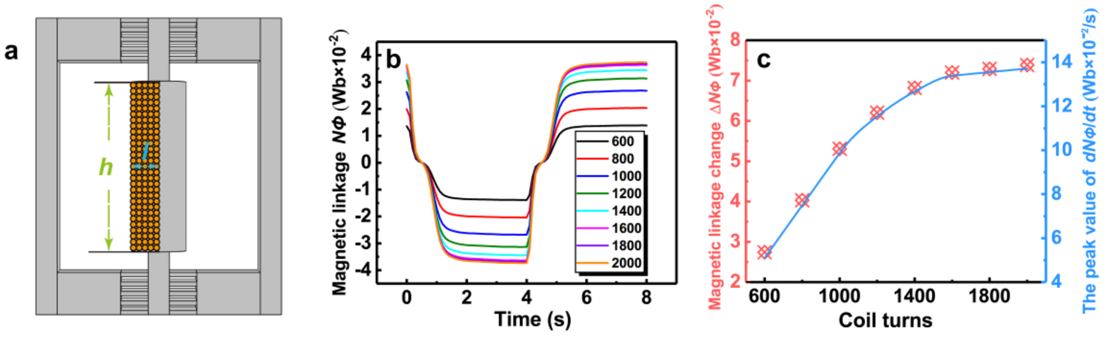

Supplementary Figure 9: The effect of coil turns on the magnetic circuit. **a**, Schematic diagram of the change of the coil. **b,** The change curve of the flux linkage in the coil with time. **c**, The magnetic linkage change $\Delta N\Phi$ and the peak value of the magnetic linkage change rate $dN\Phi/dt$ with the coil turns. (Source data are provided as a Source Data file.)

(4) Structural optimization of the magnetic circuit

Based to above results, we summarize the effects of the thickness and length of the MCS as well as the coil turns N on the magnetic linkage change rate $dN\Phi/dt$ as shown in Supplementary Fig.10. It can be seen that the $dN\Phi/dt$ increases by 65.9% with the 100% increase of thickness. However, the temperature change rate dT/dt would decrease with the increase of thickness, and so cause the decrease of $dN\Phi/dt$ as the thickness further increases. By further comparing the performance of two 2.5 mm thick samples in each sample cabin and five 1 mm thick samples in each sample cabin (Fig. 2d and Supplementary Fig. 6), we found that layered thin plates are favorable to higher TMG performance, and so 1 mm × 5 layered

plates are chosen as the MCS in the following study. The increase of length enhances the TMG performance significantly at first by reducing the shunt magnetic flux in the outer loop. Then, the permanent magnet cannot fully magnetize the FM MCS when the length is larger than 5~7 mm, and a large amount of magnetic flux is strayed into the air, leading to the remarkable decrease of $dN\Phi/dt$. Therefore, we choose 6.5 mm as the optimized length for MCS. In addition, the coil turns N plays the most impact on the TMG performance, e.g., we found that the $dN\Phi/dt$ increases largely by 83.1% with the 100% increase of N. But the surface coil might not be able to effectively sense flux changes in the center yoke when $N > 1600$, resulting in the gradual saturation of $dN\Phi/dt$. Therefore, a coil turns of 1600 is chosen as the optimized parameter. The structural parameters before and after optimization are shown in Supplementary Table 1. Based on above optimized parameters, we further optimized the magnetic circuit structure.

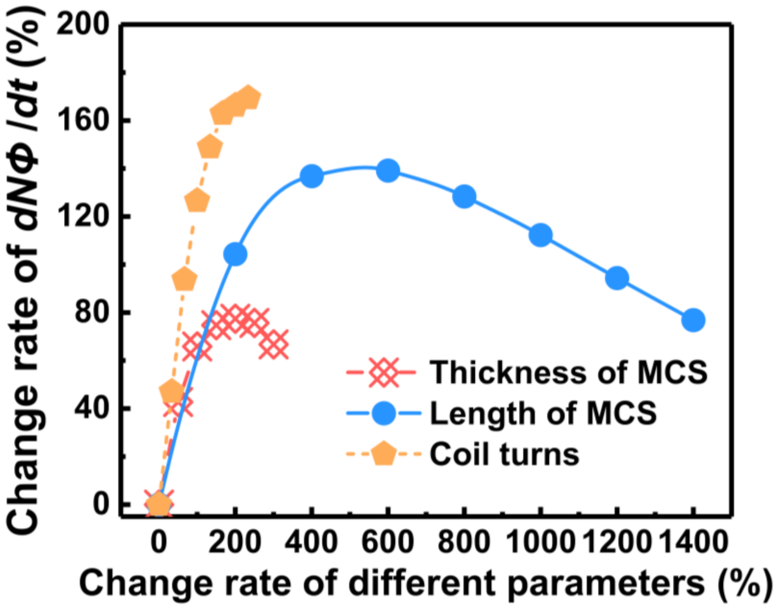

Supplementary Figure 10: The relationship between the parameter change rate and the magnetic linkage change rate $dN\Phi/dt$. (Source data are provided as a Source Data file.)

Supplementary Table 1: Structural parameters before and after the optimization.

	Before optimization	After optimization
Thickness of the MCS	1 mm × 2	1 mm × 5
Length of the MCS	10 mm	6.5 mm

Coil turns	600	1600

Supplementary Note 4. Optimization of test parameters

(1) Effect of the cold end temperature

In order to further improve the performance of our TMG, we studied the effect of the non-structural parameters on the TMG performance through a combination of experiment and finite element simulation. Supplementary Fig. 11a-e compares the *V*-*t*, *T*-*t*, **B**-*t*, and corresponding *d***B**/*dt* curves with same T_{hot} = 363 K but different T_{cold}. The peak *V* value decreases almost linearly from 0.18 to 0.03 V with increasing the cold end temperature from 278 to 298 K. In addition, the average power P_{avg} also decreases significantly from 74.5 μW to 2.5 μW with increasing cold end temperature from 278 to 298 K. It is seen that temperature of MCS increases very fast at the beginning, and then gradually reaches the stable temperature. Thus, the largest *dT*/*dt* is obtained around 1 s, and bigger temperature difference between the cold and hot end temperatures would cause the faster *dT*/*dt*. Supplementary Fig. 11f shows the *dT*/*dt* as a function of cold end temperature. The increase of cold end temperature leads to almost linearly decrease of *dT*/*dt*, which would lower the TMG performance. In addition, since T_C of Gd is 292 K, the magnetic transition cannot fully complete with the increase of cold end temperature, and even cannot occur when the cold end temperature is increased to 298 K. This fact will also reduce the Δ**B** of magnetic transition, as shown in Supplementary Fig. 11f. Consequently, because of both decreases of *dT*/*dt* and Δ**B**, the *d***B**/*dt* decreases remarkably with the increase of cold end temperature, e.g., the peak −*d***B**/*dt* value decreases from 0.81 T/s to 0.28 T/s with the cold end temperature increasing from 278 K to 298 K, leading to the lowering of the TMG performance.

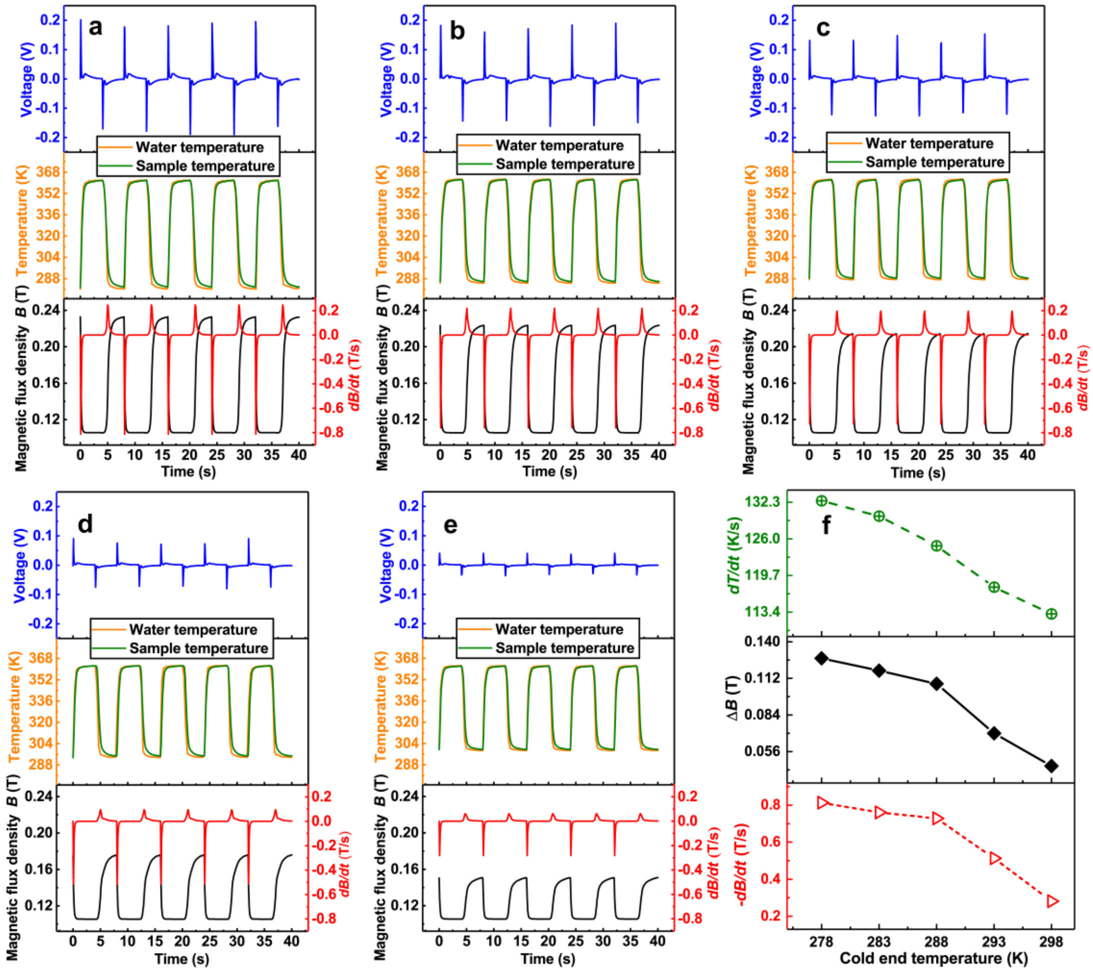

Supplementary Figure 11: The effect of cold end temperature on performance of TMG. **a**, 278 K. **b**, 283 K. **c**, 288 K. **d**, 293 K. **e**, 298 K. **f**, The curve of peak temperature change rate *dT/dt*, magnetic flux density change Δ**B**, and peak magnetic flux density change rate -*d***B***/dt* with cold end temperature. (Source data are provided as a Source Data file.)

(2) Effect of the hot end temperature

Using the same method, the effect of hot end temperature on the TMG was also studied. Supplementary Fig. 12a-e compares the *V*-*t*, *T*-*t*, *B*-*t*, and corresponding *d***B**/*dt* curves with same T_{cold} = 283 K but different T_{hot}. The peak *V* value increases from 0.13 to 0.16 V with increasing hot end temperature from 333 to 363 K. But the increasing rate is not as large as that with the decrease of cold end temperature. With further rising the hot end temperature to 373 K, the peak *V* value decreases sharply to 0.11 V. Similar to the effect of cold end temperature, bigger temperature difference between the cold and hot end temperatures would cause the faster *dT*/*dt*. Therefore, the increase of hot end temperature results in the increase

of dT/dt (Supplementary Fig. 12f). It can be seen that the peak value of dT/dt increases from 71.7 K/s to 130.0 K/s with the hot end temperature increasing from 333 K to 363 K. However, although the dT/dt increases largely with the increase of hot end temperature, the $\Delta\mathbf{B}$ does not increase very much. Since the initial hot end temperature of 333 K is much higher than T_C = 292 K, the Gd could be induced into complete PM state by the hot fluid, and the $\Delta\mathbf{B}$ during the magnetic transition already reaches the maximum. Thus, the further increase of hot end temperature could not result in a larger reduction of **B**, e.g., the $\Delta\mathbf{B}$ only increase ~0.005 T with the hot end temperature increasing from 333 K to 363 K (Supplementary Fig. 12f). Moreover, the peak value of $-d\mathbf{B}/dt$ only increases by 0.16 T/s from 0.60 T/s to 0.76 T/s with the hot end temperature increasing from 333 K to 363 K, much smaller than the increase of $|d\mathbf{B}/dt|$ (0.53 T/s) as the cold end temperature decreases from 298 K to 278 K. This fact results in the slow increase of V. When the hot end temperature further rises to 373 K, the dT/dt decreases sharply to 95.3 K/s. This is because the hot water pump reaches the highest service temperature, thus causing the flow rate to decrease from 0.06 L/s to 0.047 L/s. Then, the peak value of $-d\mathbf{B}/dt$ drops to 0.70 T/s, resulting in the reduction of TMG performance.

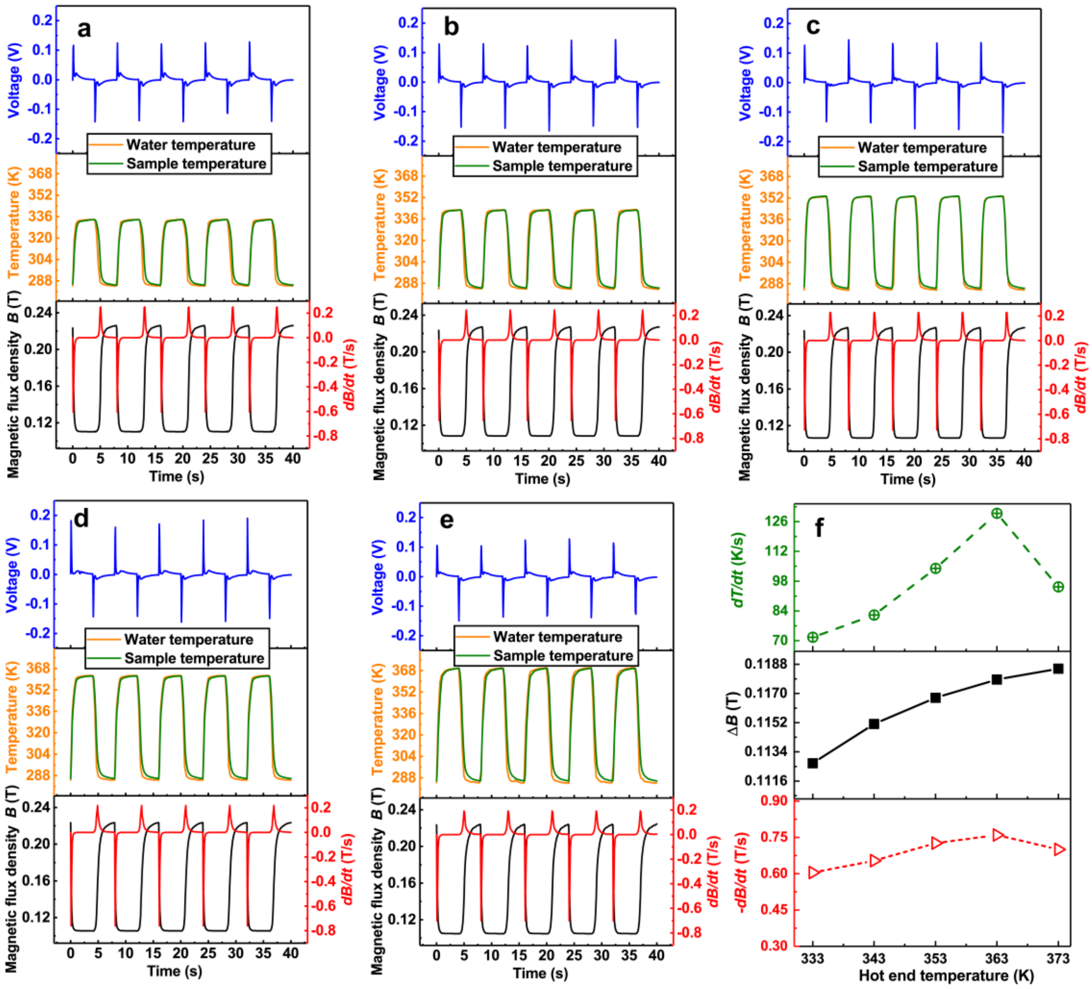

Supplementary Figure 12: The effect of hot end temperature on performance of TMG. **a**, 333 K. **b**, 343 K. **c**, 353 K. **d**, 363 K. **e**, 373 K. **f**, The curve of peak temperature change rate *dT/dt*, magnetic flux density change Δ**B**, and peak magnetic flux density change rate -*d***B**/*dt* with hot end temperature. (Source data are provided as a Source Data file.)

(3) Effect of the cycle periods

As discussed above, the temperature changes very fast at the beginning of each cycle, making the magnetic transition of MCS finish within the first few seconds. It suggests that the cycle period could be shortened to obtain more induced power. Supplementary Fig. 13a-f compares the *V*-*t*, *T*-*t*, **B**-*t*, and corresponding *d***B**/*dt* curves with different cycle periods. The peak *V* value increases gradually from 0.1 to 0.16 V with increasing the cycle period from 2 s to 8 s, and then it remains stable but the peak numbers reduce with the further increase of cycle period. It is found that a discrepancy between sample temperature and water temperature appears when the cycle period is less than 8 s, suggesting that the sample temperature cannot

reach a stable equilibrium with the heating/cooling fluid. This would cause the narrower temperature difference between the cold and hot end temperatures as well as incomplete magnetic transition. As mentioned above, the narrower temperature difference would lead to the lower dT/dt, as shown in Supplementary Fig. 14a. Meanwhile, the incomplete magnetic transition results in the reduction of $\Delta\mathbf{B}$ (Supplementary Fig. 14b). With the increase of cycle period, the discrepancy between sample temperature and water temperature disappears and a stable temperature equilibrium is reached. Thus, temperature difference between the cold and hot end temperatures increases and the magnetic transition can be completed, which lead to the increase of both dT/dt and $\Delta\mathbf{B}$. When the cycle period is larger than 8 s, both dT/dt and $\Delta\mathbf{B}$ reach the maximum. Above variation of dT/dt and $\Delta\mathbf{B}$ gives rise to similar change of corresponding $d\mathbf{B}/dt$ as shown in Supplementary Fig. 14c. The peak value of $-d\mathbf{B}/dt$ increases from 0.43 T/s to 0.76 T/s with the cycle period increasing from 2 s to 8 s, and then reaches the saturation. Although the increase of cycle period could enhance the induced V peak, the peak numbers reduce due to the longer cycle period, which is not favorable to obtaining more induced power. Therefore, it is seen from Fig. 4c that the P_{avg} increases at first by increasing cycle period from 2 s to 4 s, and then decreases distinctly with the increase of cycle period.

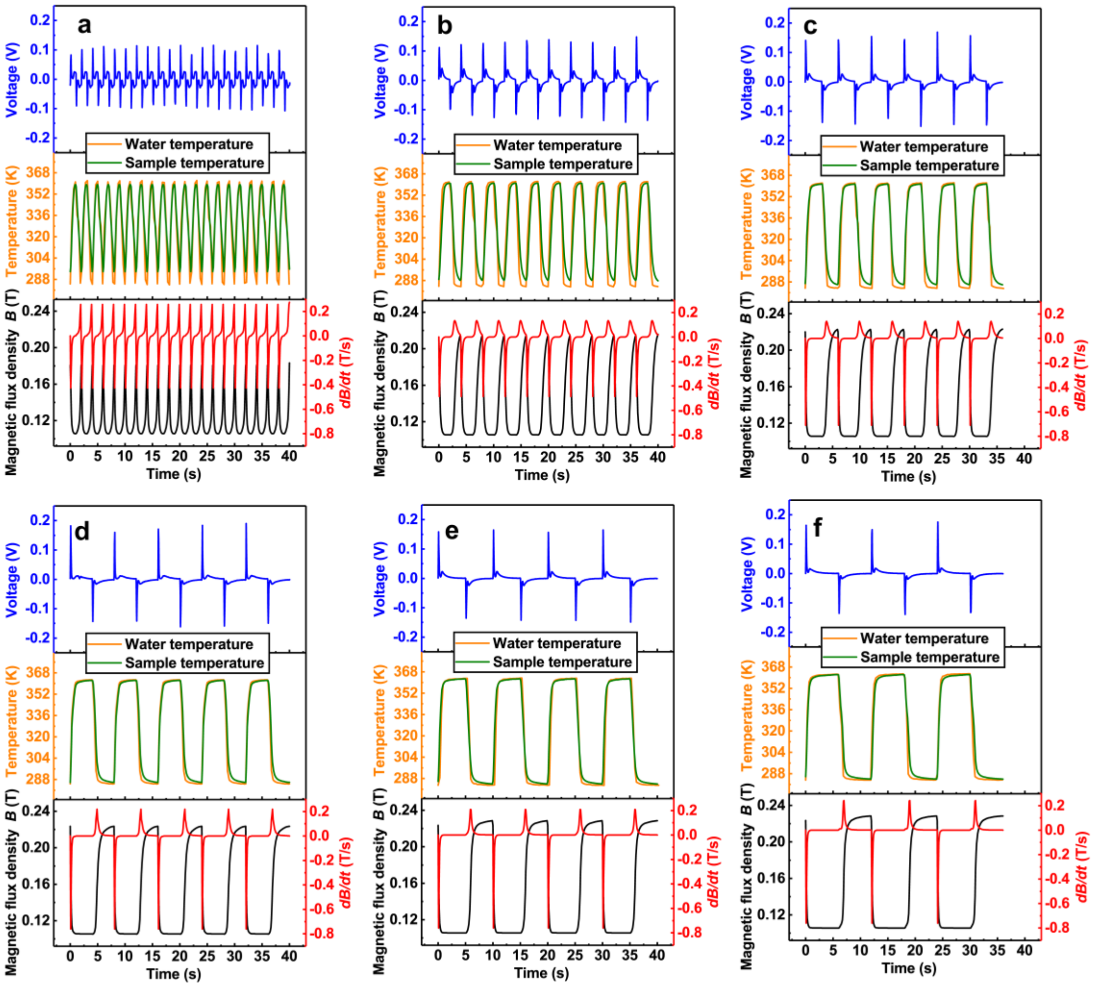

Supplementary Figure 13: The effect of the cycle periods on performance of TMG. **a**, 2 s. **b**, 4 s. **c**, 6 s. **d**, 8 s. **e**, 10 s. **f**, 12 s. (Source data are provided as a Source Data file.)

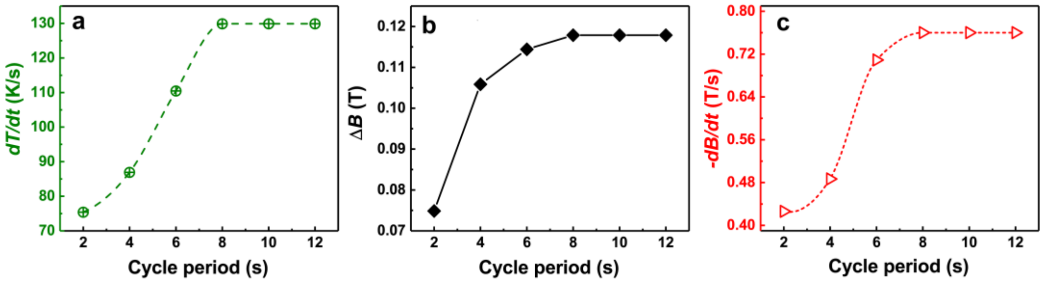

Supplementary Figure 14: a, The curve of peak temperature change rate *dT/dt* with cycle period. **b**, The curve of magnetic flux density change Δ**B** with cycle period. **c**, The curve of peak magnetic flux density change rate -*d***B**/*dt* with cycle period. (Source data are provided as a Source Data file.)

Based on the above analysis, it is found that the lower cold end temperature and higher hot end temperature are favorable to higher TMG performance. Besides, shorter cycle period

could ensure to obtain more induced V and higher P_{avg}. Accordingly, we optimized the hot end temperature, cold end temperature, and cycle period as 278 K, 363 K, and 4 s, respectively. The parameters before and after optimization are shown in Supplementary Table 2.

Supplementary Table 2: Test parameters before and after parameter optimization.

	Before optimization	After optimization
Cold end temperature	283 K	278 K
Hot end temperature	363 K	363 K
Cycle period	8 s	4 s

Supplementary Note 5. Performance test of LaFeSiH/In

$La_{0.7}Ce_{0.3}Fe_{11.51}Mn_{0.09}Si_{1.4}H$/In compound has a high MCE as well as low thermal and magnetic hysteresis, and so is a very promising TMG material. In our previous study, LaFeSiH/In composite has been demonstrated to exhibit the outstanding TMG performance among the typical MCE materials. Therefore, we further used LaFeSiH/In as MCS to test the TMG performance and compared it with Gd. Supplementary Fig. 15a shows the temperature dependence of the ZFC and FC magnetization for LaFeSiH/In under 0.2 T. The LaFeSiH/In undergoes a FM-PM magnetic transition around T_C. The T_C is determined as the minimum value of dM/dT curve as shown in the inset, and it is 310 K during the heating and 308 K during the cooling, respectively. Such small thermal hysteresis of 2 K indicates the nature of weak first-order magnetic transition (FOMT). Supplementary Fig. 15b shows the **M-H** curves of LaFeSiH/In measured in field increasing and decreasing modes around T_C. It is seen than the LaFeSiH/In exhibits a magnetic hysteresis around T_C and a sharp change in magnetization above a critical field, indicating the characteristic of the first-order itinerant-electron metamagnetic (IEM) transition. The maximum value of hysteresis loss, defined as the enclosed area between the ascending and descending magnetization curves, is obtained to be 11 J/kg. It is much lower than that of La(Fe, Si)$_{13}$ materials with typical FOMT, i.e., 158 J/kg for $La_{0.8}Ce_{0.2}Fe_{11.5}Si_{1.5}$ and 22 J/kg for $LaFe_{11.5}Si_{1.5}$[12], thus suggesting the nature of weak FOMT of our LaFeSiH/In material. As mentioned, small thermal and magnetic hysteresis is

favorable to the application of MCE and TMG during the heating and cooling cycles. Based to the **M**-**H** curves, the magnetic entropy change ΔS_M values for different magnetic field changes were calculated by using Maxwell relation, as shown in Supplementary Fig. 15c. The maximum ΔS_M are 7.41 J/kg K, 10.82 J/kg K, and 12.31 J/kg K for the magnetic field changes of 1 T, 2 T, and 3 T, respectively, which are larger than those of Gd metal. The larger MCE suggests the possible higher TMG performance of LaFeSiH/In than Gd metal. Supplementary Fig. 15d shows the temperature dependence of C_P for LaFeSiH/In. The C_P reaches the peak of ~932.8 J/kg K at T_C, and then decreases sharply with the increase of temperature, corresponding to the typical characteristic of FOMT. The C_P of LaFeSiH/In is much higher than that of Gd metal, revealing that LaFeSiH/In absorbs higher thermal energy during the phase transition process than that of Gd metal. However, the λ of LaFeSiH/In is higher than that of Gd metal, as shown in Fig. 3 from Ref. 6. It indicates that LaFeSiH/In would show faster temperature change than that of Gd metal under the same heat absorption/release conditions.

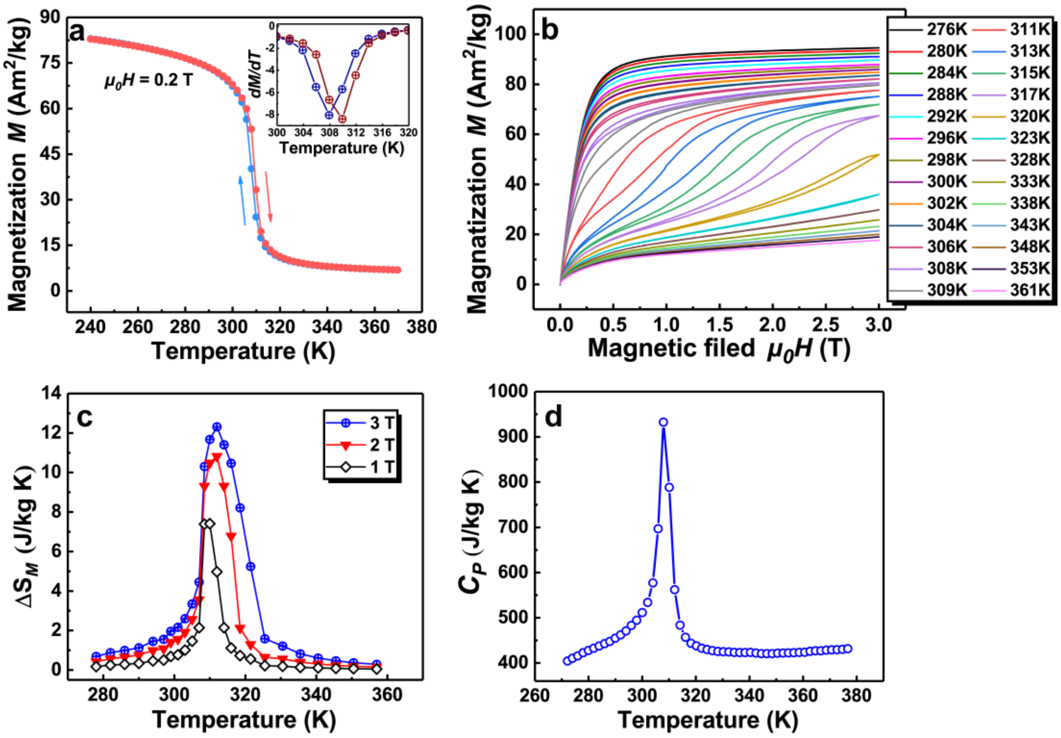

Supplementary Figure 15: Magnetic and thermal properties of thermomagnetic material LaFeSiH/In. **a**, Variation curve of magnetization **M** with temperature *T.* The inset shows the magnetization change rate *d***M**/*dT* curve. **b**, Variation curve of magnetization **M** with magnetic field **H** at different temperatures. **c**, Variation curve of magnetic entropy change ΔS_M under

different magnetic field changes. **d**, Variation curve of specific heat capacity C_P with temperature T. (Source data are provided as a Source Data file.)

Based on the optimized test parameters, we tested the thermomagnetic power generation performance of the LaFeSiH/In. Supplementary Fig. 16 compares the V-t, T-t, **B**-t, and corresponding $d\mathbf{B}/dt$ curves for (a) LaFeSiH/In and (b) Gd. It is noted that the induced V is divided by the sample mass for a more reasonable comparison. Similar to the case of Gd, two successive induced V peaks are also obtained by LaFeSiH/In since both LaFeSiH/In MCSs on both sides are not turned ON/OFF at the same time. A big V peak of 0.02 V/g is induced by heating the right MCS to OFF at ~0.2 s, and then a small V peak of 0.01 V/g is followed by cooling left MCS to ON at ~0.7 s. In comparison, the TMG using Gd metal as MCS generates a big V peak of 0.031 V/g at ~0.1 s and a small V peak of 0.007 V/g at ~0.8 s. In addition, it is found that the induced V during the heating is much larger than the one during the cooling. It is seen from the T-t curves that both T_Cs of LaFeSiH/In and Gd are close to the cold end temperature. So magnetic transition would occur earlier during the heating than that during the cooling. Since the temperature changes fast at the beginning of heating/cooling process due to the big difference between the sample and water temperatures, the dT/dt around T_C during the heating is larger than that during the cooling. Therefore, it causes the larger $d\mathbf{B}/dt$ peak during the heating than the one obtained during the cooling, and correspondingly, the induced V during the heating is much larger than the V during the cooling. In addition, it is found that the V peak of 0.02 V/g during heating for LaFeSiH/In is lower than the one (0.031 V/g) for Gd. But the V peak of 0.01 V/g during cooling for LaFeSiH/In is slightly higher than the value of 0.007 V/g for Gd. It is seen from the **M**-T curves (Supplementary Fig. 1a and Supplementary Fig. 15a) that the $d\mathbf{M}/dT$ of LaFeSiH/In is ~8.4 Am^2/kg K, which is more than twice the $d\mathbf{M}/dT$ of ~3.5 Am^2/kg K for Gd metal. This suggests that the LaFeSiH/In has a higher $d\mathbf{B}/dT$ than that of Gd during both heating and cooling. As shown in Supplementary Fig. 16, the T_C of Gd is closer to the cold end temperature than the T_C of LaFeSiH/In. Therefore, the dT/dt around T_C for Gd is larger than that for LaFeSiH/In during heating, while it is lower than the dT/dt for LaFeSiH/In during cooling. Consequently, according to $d\mathbf{B}/dt = (d\mathbf{B}/dT)(dT/dt)$, the $d\mathbf{B}/dt$ of LaFeSiH/In during heating is −0.47 T/s, lower than the $d\mathbf{B}/dt$ of −0.76 T/s for Gd. During the cooling, the $d\mathbf{B}/dt$ of LaFeSiH/In is 0.23

T/s, ~35.2% higher than 0.16 T/s of Gd. This fact results in the different differences of induced *V* for LaFeSiH/In and Gd metal during the heating and cooling cycles.

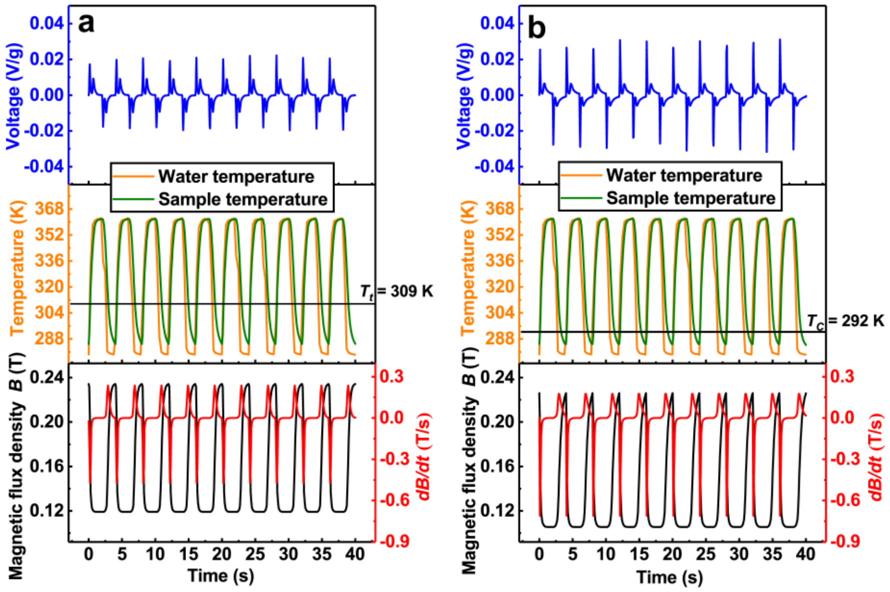

Supplementary Figure 16: The power generation performance of the two TMMs. **a**, LaFeSiH/In. **b**, Gd. (Source data are provided as a Source Data file.)

In our previous work, the location of T_C in the working temperature range influences significantly the TMG performance by affecting the dT/dt around T_C[10, 13]. Therefore, it is necessary to ensure the T_C of different materials locate at the same position of their respective working temperature range in order to compare the intrinsic TMG performance of different materials. Taking Gd as the reference whose T_C is 14 K higher than the cold end temperature, we assume to shift the working temperature range of LaFeSiH/In from 278~363 K to 295~380 K, so that the T_C of LaFeSiH/In is also 14 K higher than the cold end temperature. Supplementary Fig. 17a displays the **M**-*T* curves under 0.2 T for LaFeSiH/In and Gd metal in their respective working temperature range. It is seen that the T_Cs of both materials locate at the same position in their respective temperature range, suggesting that the dT/dt would not be affected by the extrinsic factor of T_C location. Besides, it is noted that the $d\mathbf{M}/dT$, as an intrinsic property, will not be changed by shifting the working temperature range. Supplementary Fig. 17b shows the *V*-*t*, *T*-*t*, **B**-*t*, and corresponding $d\mathbf{B}/dt$ curves for LaFeSiH/In after shifting the working temperature range. A big *V* peak of 0.034 V/g followed by a small *V* peak of 0.005 V/g are obtained after temperature range shifting. In comparison

with the original values, the big V peak induced during heating increases while the small V during cooling decreases. This is because the T_C moves closer to the cold end temperature, which would enhance the dT/dt around T_C during the heating but lower the dT/dt at T_C during cooling. Accordingly, the $|d\mathbf{B}/dt|$ during the heating increases from 0.47 T/s to 0.80 T/s, while $|d\mathbf{B}/dt|$ during the cooling decreases from 0.23 T/s to 0.12 T/s. Therefore, the V peak induced during heating increases but the V peak during cooling decreases based on the Faraday's law.

Finally, the corresponding power density P_D and relative conversion efficiency η_{rel} are calculated based on the above intrinsic TMG performance. The η_{rel} is calculated by the following formula[14]:

$$\eta_{rel} = \frac{\eta_{abs}}{\eta_{carnot}} = \frac{\eta_{abs}}{1 - \frac{T_{Cold}}{T_{Hot}}} \tag{7}$$

$$\eta_{abs} = \frac{E_M}{Q_{in}} = \frac{\mu_0 \int \mathbf{H} d\mathbf{M}}{\int C_P(T) dT} \tag{8}$$

where η_{abs} is the absolute efficiency corresponding to the conversion rate of thermal energy to magnetic energy, η_{Carnot} is Carnot efficiency $(T_{Hot}-T_{Cold})/T_{Hot}$, T_{Cold} is the cold end temperature, T_{Hot} is the hot end temperature, E_M is the magnetic energy output by the MCS during the cycle, which is the upper limit of the energy output of the TMG, Q_{in} is the heat absorbed by the TMM during the cycle, **M** is the magnetization of the MCS, respectively.

Supplementary Fig. 17c compares the key TMG factors of Gd and LaFeSiH/In materials. Except the relatively lower conversion efficiency η_{rel}, the peak voltage of 0.034 V/g, the max power density P_{Dmax} (power divided by the material volume) of 4 mW/cm^3, and the average power density P_{Dave} of 232 μW/cm^3 for LaFeSiH/In are much higher than those of Gd (0.031 V/g, 3.2 mW/cm^3, and 156 μW/cm^3). The main reason for the lower η_{rel} is possibly due to the higher C_P of LaFeSiH/In which results in the higher Q_{in}. Overall, it can be seen that LaFeSiH/In exhibits more excellent comprehensive properties than Gd metal.

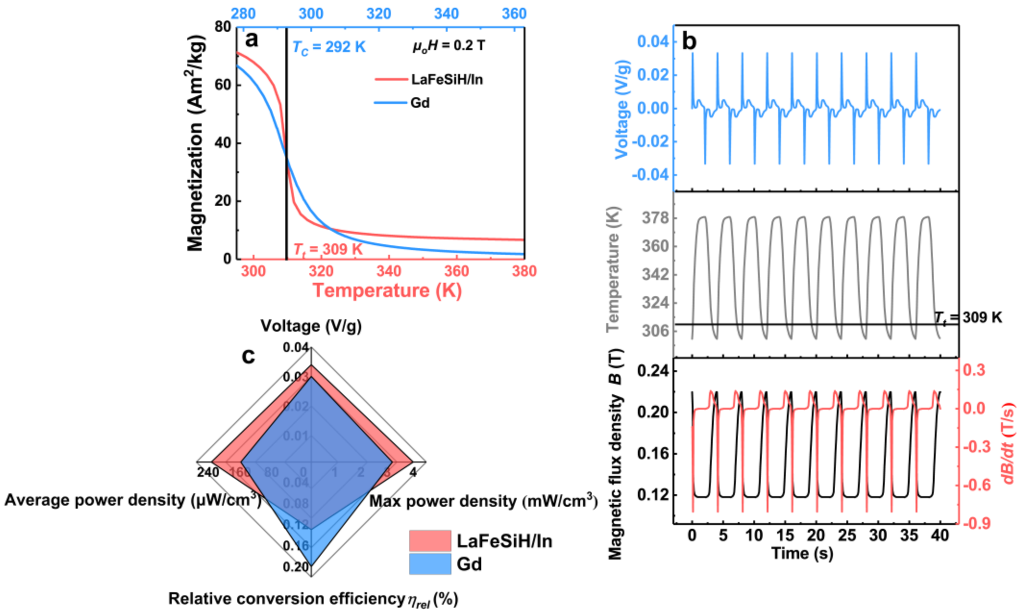

Supplementary Figure 17: **a**, The **M**-*T* curves for LaFeSiH/In and Gd after shifting the working temperature range. **b**, The *V*-*t*, *T*-*t*, **B**-*t*, and corresponding *d***B**/*dt* curves for LaFeSiH/In after shifting the working temperature range. **c**, The comprehensive comparison of these two materials. (Source data are provided as a Source Data file.)

Supplementary Note 6. The circuit for lighting up the LEDs

By connecting the TMG with LEDs, our TMG successfully lights up the LEDs. Supplementary Fig. 18 shows the circuit schematic for lighting up the LEDs. The power generated by the TMG is amplified by an amplify module and then lights up the LEDs. Due to the change in the direction of the voltage signal, the two diodes flash respectively, i.e., the yellow LED is lit when the TMG generates positive voltage, while the red LED is lit when a negative voltage is generated (Fig. 5d of the main paper). In addition, due to the successive big and small *V* peaks during each heating/cooling process, the LED will flash twice with different brightness. The process of LED lighting is shown in the Supplementary Movie 1.

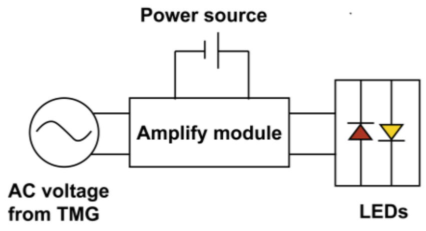

Supplementary Figure 18: Circuit schematic for lighting up LEDs.

Supplementary References

1. Hansen, B. R. et al. Properties of magnetocaloric La(Fe, Co, $Si)_{13}$ produced by powder metallurgy, *J. Magn. Magn. Mater.* **322**, 3447-3454 (2010).
2. Wang, Y. X. et al. Outstanding comprehensive performance of La(Fe, $Si)_{13}H_y$/In composite with durable service life for magnetic refrigeration, *Adv. Electron. Mater.* **4**, 1700636 (2018).
3. Ou, Y. Y. et al. Plastically deformed La-Fe-Si: Microstructural evolution, magnetocaloric effect and anisotropic thermal conductivity, *Acta. Mater.* **187**, 1-11 (2020).
4. Batdalov, A. B. et al. Magnetic, thermal, and electrical properties of an $Ni_{45.37}Mn_{40.91}In_{13.72}$ Heusler alloy, *J. Exp. Theor. Phys.* **122**, 874-882 (2016).
5. Qu, Y. H. et al. Giant and reversible room-temperature magnetocaloric effect in Ti-doped Ni-Co-Mn-Sn magnetic shape memory alloys, *Acta. Mater.* **134**, 236-248 (2017).
6. Fukamichi, K., Fujita, A. & Fujieda, S. Large magnetocaloric effects and thermal transport properties of $La(FeSi)_{13}$ and their hydrides, *J. Alloys Compd.* **408**, 307-312 (2006).
7. Fujieda, S., Hasegawa, Y., Fujita, A. & Fukamichi, K. Thermal transport properties of magnetic refrigerants $La(Fe_xSi_{1-x})_{13}$ and their hydrides, and $Gd_5Si_2Ge_2$ and MnAs, *J. Appl. Phys.* **95**, 2429-2431 (2004).
8. Waske, A. et al. Energy harvesting near room temperature using a thermomagnetic generator with a pretzel-like magnetic flux topology. *Nat. Energy* **4**, 68-74 (2019).
9. Brillouin, L. & Iskenderian, H. P. Thermomagnetic generator. *Electr. Commun.* **25**, 300-311 (1948).
10. Ma, Z. H. et al. Thermomagnetic generation performance of Gd and La(Fe, $Si)_{13}H_y$/In material for low-grade waste heat recovery. *Adv. Sustain. Syst.* **5**, 2000234 (2021).
11. Deepak, K., Varma, V. B., Prasanna, G. & Ramanujan, R. V. Hybrid thermomagnetic oscillator for cooling and direct waste heat conversion to electricity. *Appl. Energy* **233-234**, 312-320 (2019).

12. Zhang, H. et al. Simultaneous enhancements of Curie temperature and magnetocaloric effects in the $La_{1-x}Ce_xFe_{11.5}Si_{1.5}C_y$ compounds. *J. Magn. Magn. Mater.* **324**, 484-487 (2012).
13. Chen, H. D. et al. Evaluation of thermomagnetic generation performance of classic magnetocaloric materials for harvesting low-grade waste heat. *Appl. Energy* **306**, 1-10 (2022).
14. Dzekan, D., Waske, A., Nielsch, K. & Fahler, S. Efficient and affordable thermomagnetic materials for harvesting low grade waste heat. *APL Mater.* **9**, 011105 (2021).
