# Supplementary material for: High-performance thermomagnetic generator controlled by a magnetocaloric switch
Source: Nat Commun. 2023 Aug 9;14:4811. doi: 10.1038/s41467-023-40634-x (PMC10412618; doi:10.1038/s41467-023-40634-x)
Supplement: Supplementary file 3 — Description of Additional Supplementary Files [file 41467_2023_40634_MOESM3_ESM.pdf]

Description of Additional Supplementary Files

File Name: Supplementary Movie 1
Description: Lighting up a LED with the thermomagnetic generator controlled by a magnetocaloric switch
